# Supplementary material for: Quantum electrodynamics at room temperature coupling a single vibrating molecule with a plasmonic nanocavity
Source: Nat Commun. 2019 Mar 5;10:1049. doi: 10.1038/s41467-019-08611-5 (PMC6400948; doi:10.1038/s41467-019-08611-5)
Supplement: Supplementary file 1 — Supplementary Information [file 41467_2019_8611_MOESM1_ESM.pdf]

Supplemental Information

Quantum electrodynamics at room temperature coupling a single vibrating molecule with a plasmonic nanocavity

Oluwafemi S. Ojambati, Rohit Chikkaraddy, William Deacon, Matthew Horton, Dean Kos, Vladimir A. Turek, Ulrich Keyser, Jeremy J. Baumberg

NanoPhotonics Centre, Cavendish Laboratory, Department of Physics, JJ Thompson Avenue, University of Cambridge, Cambridge, CB3 0HE, United Kingdom

Supplementary Note 1: Sample preparation

A detailed description of the sample fabrication and characterization are reported in [1]. In brief, a 2-layer plate-like DNA origami of dimensions 55x50x5 nm was designed with the software *cadnano*[2] to include 6 (10xPolyA) overhangs (complementary to thiolated ss-DNA functionalized NPs) arranged in a hexagon with diameter 10 nm, and a single 3' Atto647 modified staple in the center of the overhangs. After folding using a 10 fold excess of staples to scaffold in a 14 mM $MgCl_2$ 1x TE buffer using a 23 hour 95 → 4 ^oC temperature ramp, the origami was washed and left to functionalise on template stripped gold overnight at room temperature in an 11 mM $MgCl_2$ 0.5xTBE buffer. These gold samples were then washed with water and finally the DNA functionalized NPs were allowed to hybridize to the origami in an 11 mM $MgCl_2$ 0.5xTBE buffer at room temperature for 30 minutes, prior to subsequent washing and drying under nitrogen. The samples are characterized by AFM, TEM and DF scattering as described in [1].

Supplementary Note 2: Changes in dark-field scattering spectra

In Fig. 1b, we show the darkfield spectra evidence a consistent red-shift of 20 nm when a single Atto647 dye molecule is embedded inside the cavity. In both cases the same DNAo, same NPs, and same conditions are used. We comment here on the possible reasons for this observation. We start by calculating the coupled plasmon mode area A_p which is given by $A_p = Dd/n_D^2$ (see Ref.[3]), where D is the nanoparticle diameter, d is the gap size, and $n_D = 2.1$ is the measured refractive index of DNAo in these structures (see [1], and comment below) so we obtain $A_p = 36\ \mathrm{nm}^2$. Using an effective area for each dye molecule of $A_d \approx 1\ \mathrm{nm}^2$ and the on-resonant refractive index of a single dye molecule as $n_d \approx 4$ (see Ref.4), the effective refractive index in the gap n_g can be estimated as

$$n_g = n_D + (n_d - n_D)\frac{A_d}{A_p}. \tag{1}$$

The estimated n_g corresponds to an increase in effective gap refractive index of $\Delta n = 5\%$. Using a simple 2D Fabry-Perot resonator model for the coupled plasmon resonance, based on nanoparticle facet width w we get discrete wavelengths supported by the gap[5,6]

$$\lambda_c = \lambda_p \sqrt{\frac{w n_g^2}{d\alpha_1} + \varepsilon_\infty} \tag{2}$$

where α_1 is the first zero of the Bessel function, and λ_p is the plasma wavelength for Au ~148nm with background permittivity ε_∞. The fractional change in wavelength is then

$$\frac{\delta\lambda_c}{\delta n_g} = \frac{\lambda_c}{n_g}\left(\frac{\lambda_p}{\lambda_c}\right)^2 . n_g^2 \frac{w}{d\alpha_1} \quad (3)$$

From Eq. (S3), we obtain a fractional change in wavelength of ~2%, which is close to the observed 3% wavelength redshift. Therefore, the observed shift in the cavity resonance is indeed plausibly due to the change in effective refractive index of the gap. Such shifts are also expected from the approach of strong-coupling in the regime utilised here, since exactly the change in round trip phase in the nanocavity is responsible for strong coupling and shifts the coupled mode plasmon.

We note the refractive index of the DNA origami comes from previous papers [37,39,41] which extract it from careful fitting of coupled mode wavelengths (ie. in close proximity to Au surfaces). Additional contributions from other effects such as facet size, uncertainty in gap size, and screening effects due to free electrons in NP, are beyond the scope of this paper.

Supplementary Note 3: Surface-Enhanced Raman Scattering (SERS) from Atto647 in Au aggregate

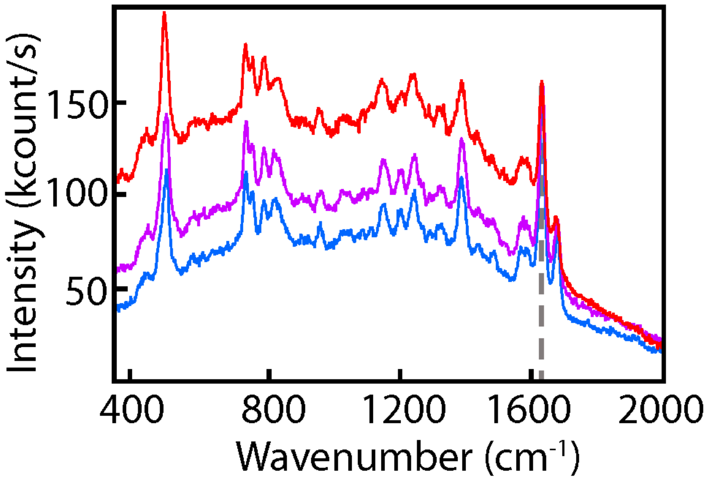

Supplementary Figure 1: Raman (SERS) and fluorescent emission from Atto647 combined into 80 nm Au nanoparticle aggregates vs wavenumber with an excitation wavelength of 633 nm. The different plots are different positions on the aggregate. The gray dashed line indicates $1625\ \mathrm{cm}^{-1}$ phonon, which corresponds to the $1a$ to $0a$ transition. Signals below $400\ \mathrm{cm}^{-1}$ are blocked by a longpass spectral filter.

Supplementary Note 4: Power dependence of emitted light from Atto647 in NPoM

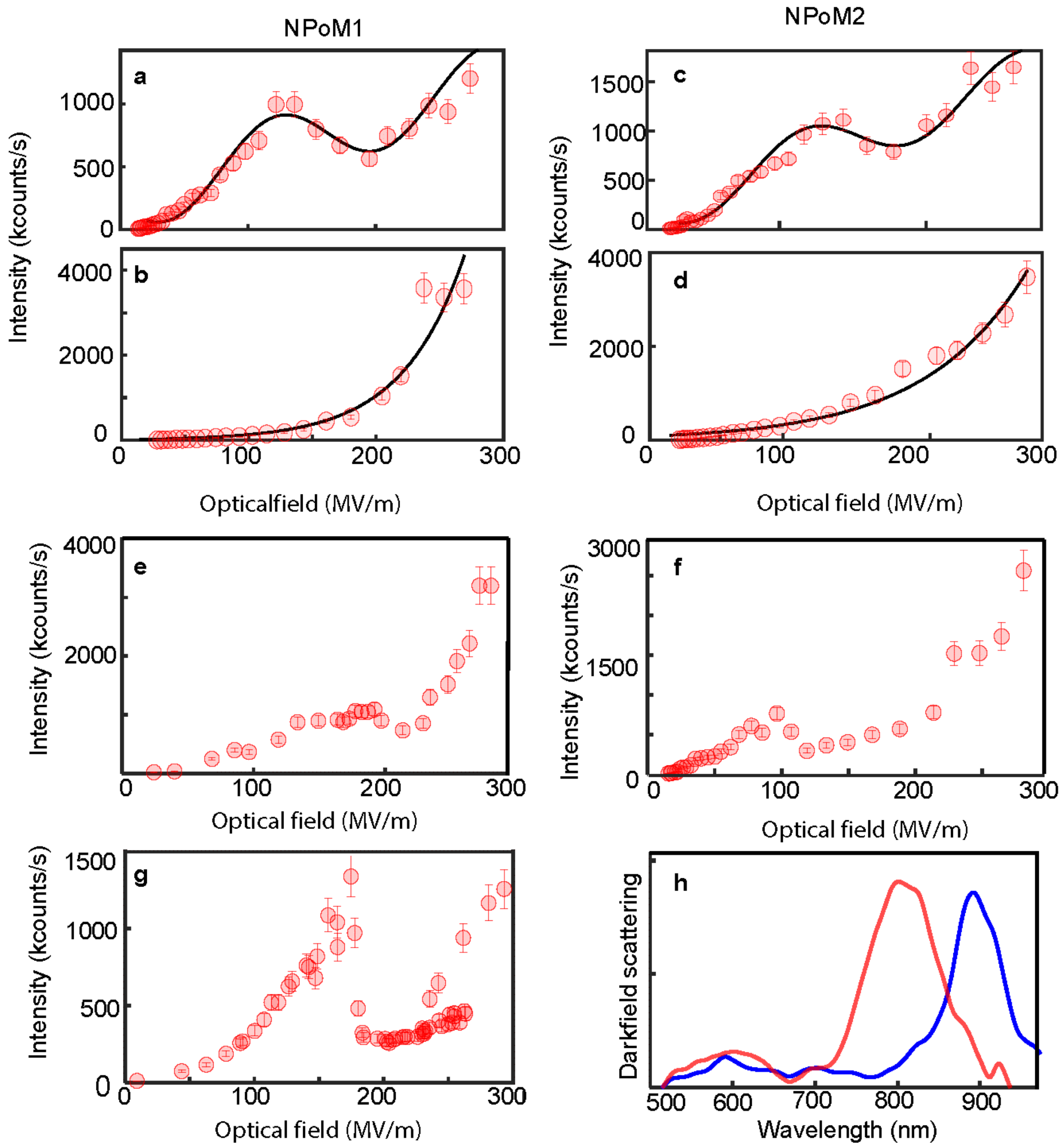

Supplementary Figure 2: Measured counts vs pulsed excitation power for single Atto647 molecules in two NPoMs. (a,b) show the first run of each experiment from low power to high power. (c,d) show the second run from low power to high power again, after permanent Au atom movement on the nanoparticle facet which red-shifts the plasmon. (e-g) Similar measured counts *vs* pulsed excitation power for several different nanocavities. (h) Darkfield scattering before (red) and after (blue) power series scan in (g). Error bars are the fluctuations of the measured counts on the detector.

The Rabi oscillations are described using the probability that an electron is left in the excited state $|c(t)|^2$ and is given as [7,8]

$$|c(t)|^2 = \frac{1}{2(1+2\xi^2)}\left[1-\left(\cos\Omega'\ t+\frac{3\xi}{\sqrt{4-\xi^2}\sin\Omega'\ t}\right)\exp\left(-\frac{3\gamma t}{2}\right)\right] \quad (4)$$

where $\xi = \frac{\gamma}{\Omega_R}$, $\Omega' = \Omega_R \sqrt{1 - \frac{\xi^2}{4}}$, t is the time, γ is the damping rate, Rabi frequency $\Omega_R = |\mu_{12} E_0 / \hbar|$, μ_{12} is the transition dipole moment, E_0 is the electric field amplitude, and $\hbar$ is Planck's constant. The pulse area Θ is then calculated using [8]

$$\Theta = \left| \frac{\mu_{12}}{\hbar} \int_0^T E_0(t) dt \right| \tag{5}$$

where T is the pulse duration.

We note that these oscillations are frequently lost on the second experimental run on the same NPoM (as shown in Supplementary Figure 2) because the plasmon cavity mode is shifting from the movement of Au atoms (see [49,50]). Although some emission is still seen, it is no longer in resonance, as clearly observed in Fig.3a. The molecule is surprisingly robust even at higher excitation powers (see Supplementary Figure 3), since there is no observable step-wise decrease in intensity, which would be obtained from bleaching. A slower decrease in intensity is observed which is a result of the red-shift of the cavity resonance that is detuned from the molecule emission peak and the excitation overlap with the pump wavelength.

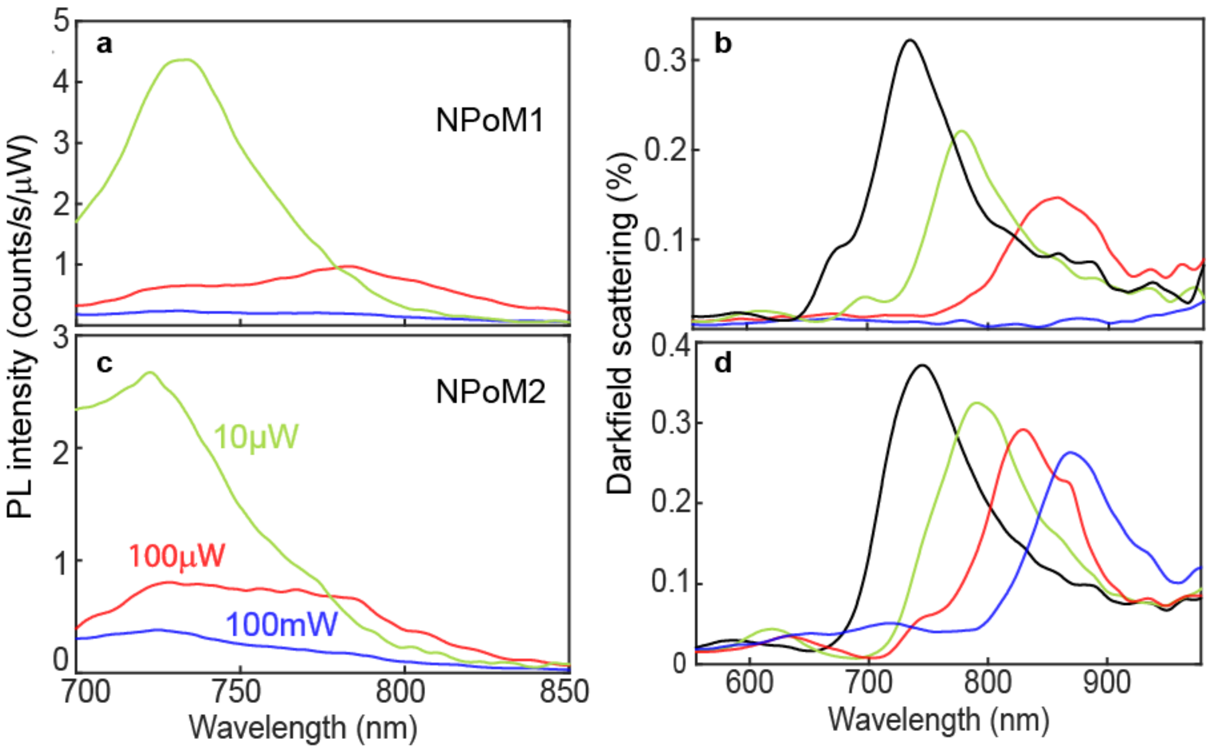

Supplementary Figure 3: (a,c) Photoluminescence spectra of two NPoMs for different excitation pulse powers. (b,d) Darkfield scattering spectra after each power in (a) and (c). The black curve is the initial scattering spectra before the PL measurements at increasing power.

Supplementary Note 5: Emission spectrum of Atto647 in NPoM

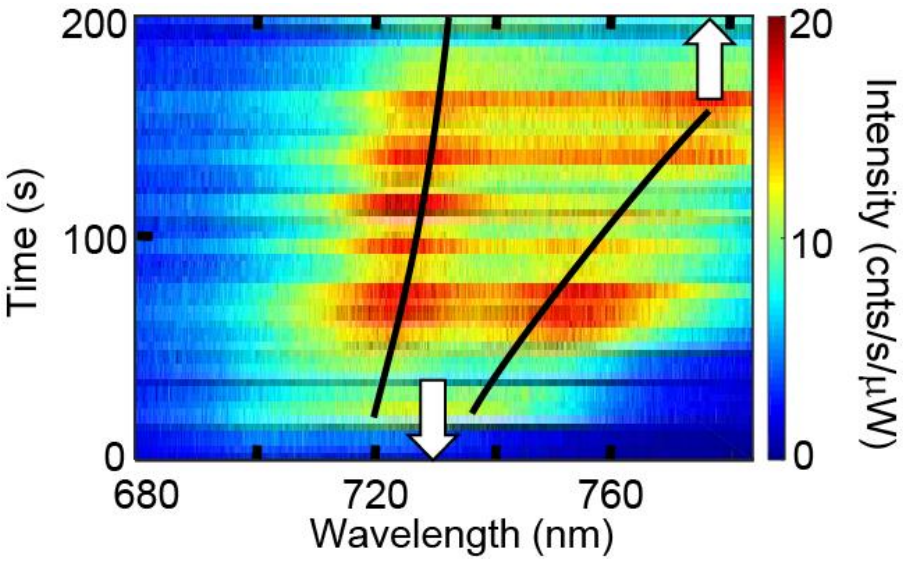

Supplementary Figure 4: Color map of photoluminescence spectra of emission of Atto647 in NPoM. Spectra are specific times are already shown in Fig. 3a.

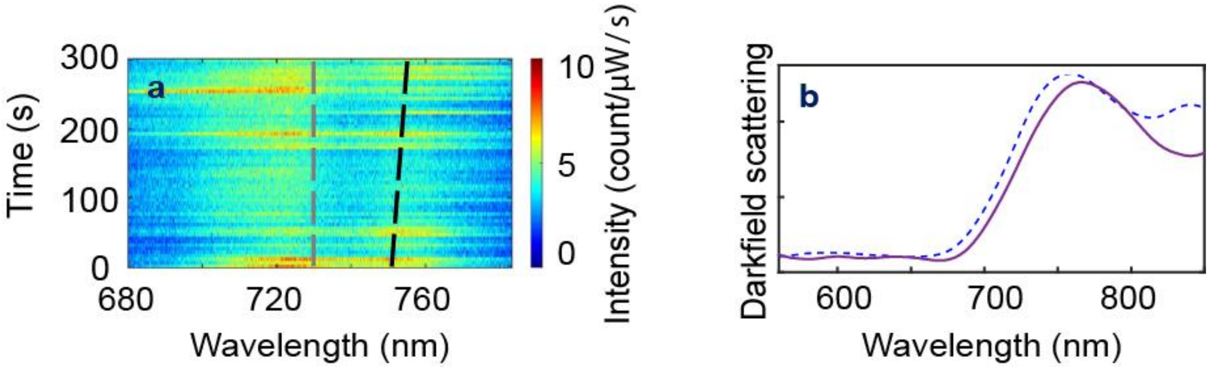

Supplementary Figure 5:(a) Time evolution of the emission from a different single Atto647 in NPoM, which in this case shows minimal DF red-shifts with irradiation time. Grey and black dashed lines show the emission peak and cavity resonances, respectively. (b) Dark field scattering spectra before (dashed blue) and after (solid purple) the measurements in (a), showing much less red-shifting from Au atom movement.

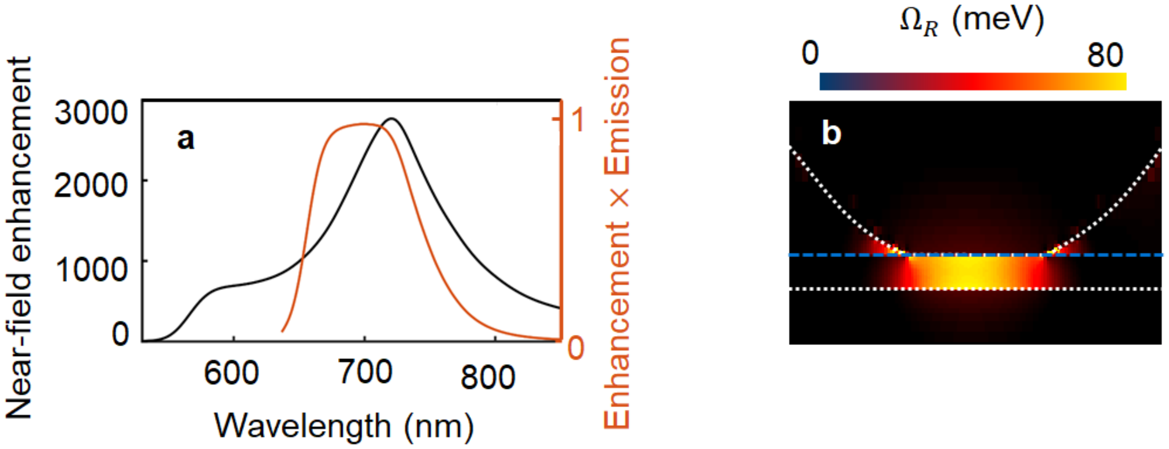

Supplementary Figure 6:: (a) Simulated near-field enhancement (solid black), with the product of

the enhancement and the emission spectra (solid orange) vs wavelength. (b) Full-wave simulation of a vertical dipole at the center of the gap with an 80 nm Au nanoparticle.

Supplementary Note 6: Second-order intensity correction $g^2(0)$: measurement and simulation

CW excitation cannot be used for the g^2 measurements as it would show a deep dip with only a sub-100fs recovery time. Since single-photon detection averages >0.3ns, then this dip would be electronically smoothed out by 0.3ns/100fs~3000 times, making the dip now 3000x broader and 3000x smaller and impossible to see over the typical noise. Instead pulsed excitation has to be used, with spacings in time larger than this instrumental time resolution. The 100fs excitation needs to match the emission time (which is <1ps from the Purcell enhancements measured) to avoid reexcitation of the dye within a single pulse.

Measurements at 610 nm:

As described in the main manuscript, we performed correction measurements at two different excitation wavelengths: 520 nm and 590 nm. We also performed the measurement at 610 nm with an input power $\sim 2\ \mu W$. We detect light from 650 nm to longer wavelengths. The result shown in Supplementary Figure 7 reveals that the detected count is over one order of magnitude higher for 610 nm pump than at the other two excitation wavelengths. The reason for this higher signal is because the excitation wavelength is closer to the absorption peak, which is at 645 nm (see Fig. 1c in the main manuscript), therefore the emitted intensity increases and the SERS signal collected also increases. As a result of a high contribution by the SERS signal, the $g^2(0)$ quickly tends

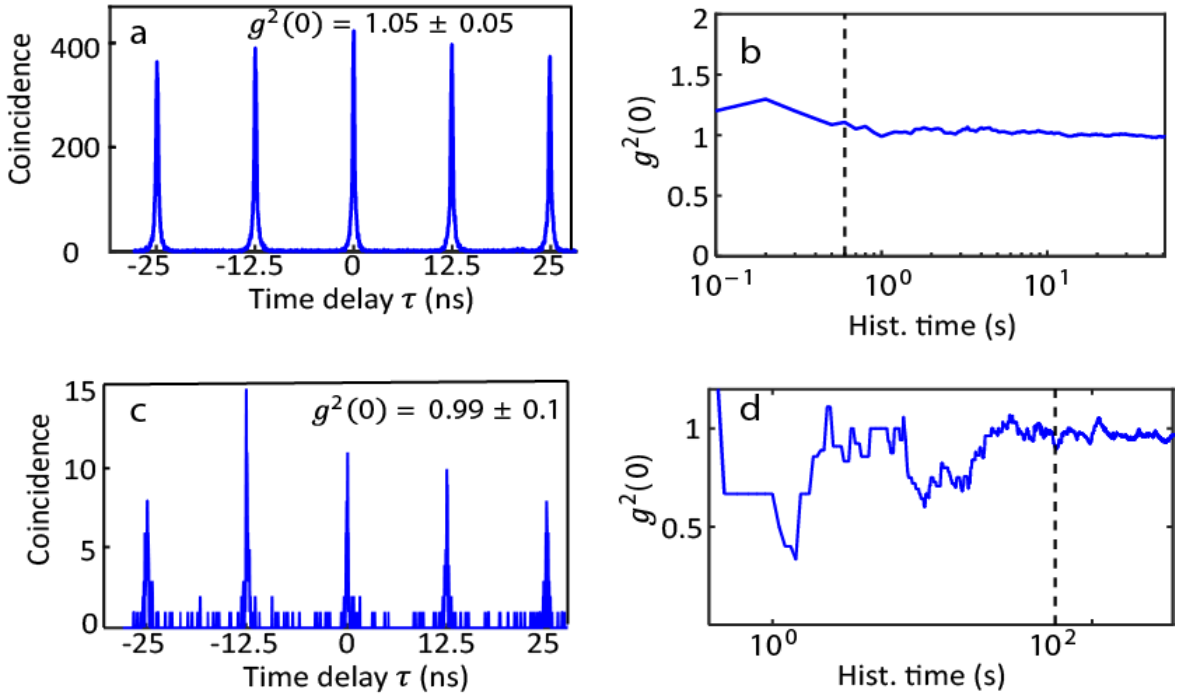

to 1 (Supplementary Figure 7b), which is expected for an uncorrelated light source.

Supplementary Figure 7:(a,c) Second-order intensity correlation (a) with the dye present at 610 nm excitation wavelength, and (c) without the dye at 590 nm excitation. (b,d) Evolution of $g^2(0)$ vs

measurement time corresponding to (a,c). Black dashed line shows time at which the upper panels are taken.

Simulation of detection efficiency on background emission:

In order to estimate the influence of the extra uncorrelated SERS-enhanced metallic emission on the estimates of $g^2(0)$, we perform numerical simulations of photon statistics to generate arrival times of photons and their correlations. Arrival times of uncorrelated photons are generated using a Poissonian distribution with a mean $\lambda{\sim}1$. For single photons (sub-Poissonian light), we use the binomial distribution to generate the arrival times. Bunched photons (e.g. thermal or chaotic light) can be described by a super-Poissonian distribution that has a variance greater than the mean. An example of such is Bose-Einstein distribution, which only applies to a single mode of a radiation field and tends to the Poissonian distribution at large numbers of modes[8]. For each of the three photon statistics regimes here, we randomly draw 100 photons from the respective distributions and simulate the procedures of the start-stop experiment: The stream of randomly generated photons is equally split into two and the time delay between the two streams of photons are computed. In our simulation of start-stop experiments, arrival times with more than two photons are counted as one photon only ignoring the rest because of the dead-time of the detectors. We obtained $g^2(0)$ from the histogram of the time delays, as explained in the main manuscript and then repeat this procedure 5000 times. We vary the fraction of uncorrelated photons δ, such that when $\delta = 0$, the generated photon distribution is entirely the correlated photon component and for $\delta = 1$, the generated photons are entirely uncorrelated, giving $g^2(0) = 1$. For $0 < \delta < 1$, the number of uncorrelated photons are 100δ and the rest are correlated photons, therefore the total number of photons is constant. In addition, at $\delta = 0$, we used different initial values of $g^2(0)$ by changing the mean λ (from 0.3 to 1) of the Bose-Einstein distribution for bunched photons. For anti-bunched photons, we introduced a fraction of uncorrelated photons to the correlated photons at $\delta = 0$ as this would be the case from multiple excitations within a pulse, so that $g^2(0) > 0$ at $\delta = 0$.

In Supplementary Figure 8(a,b) we show the results of these simulations. In the green curve of Supplementary Figure 7(a), $g^2(0)$ increases from zero (an ideal photon source) to one as the contribution of the uncorrelated photons δ increases. When $g^2(0) \neq 0$ at $\delta = 0$, $g^2(0)$ approaches one at different rates depending on the initial value of $g^2(0)$.

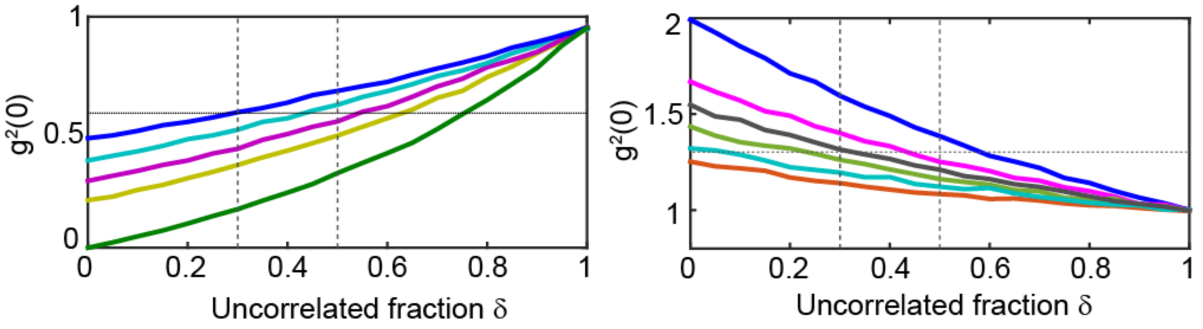

Supplementary Figure 8: (a,b) Simulated $g^2(0)$ vs contribution of uncorrelated extra background. The different colors indicate different initial $g^2(0)$ when there is no uncorrelated background. The vertical dashed gray lines bracket the estimate background contribution in the experiment, while the horizontal black dashed line is the measured $g^2(0)$ in the experiment.

From the measured $g^2(0) = 0.6$ and the background in the experiment (30 – 50%), we estimate the corrected $g^2(0) = 0.4 \pm 0.5$. For bunched photons, for different initial $g^2(0)$ at $\delta = 0$ and we find that $g^2(0)$ converges to one in all cases (Fig.S7b). Using the results of these simulations to account for the effect of the background, our measured $g^2(0)$ gives a corrected $g^2(0) = 1.45 \pm 0.5$.

Supplementary Note 7:: Measurement setup

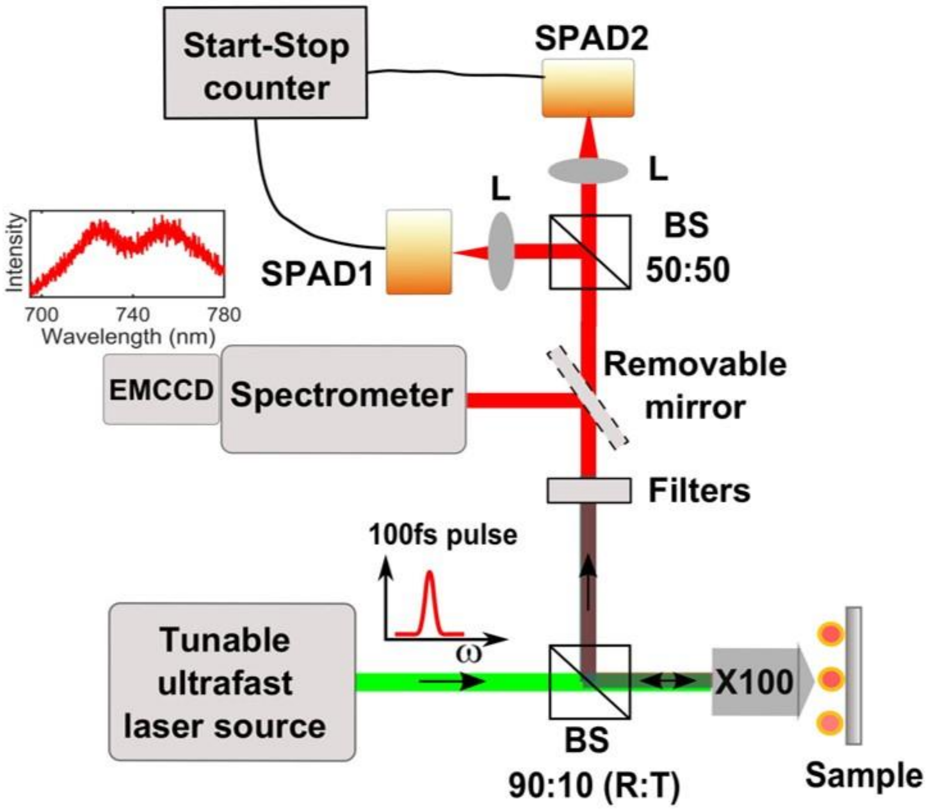

Supplementary Figure 9:: Experimental setup: A tunable laser source (500 nm – 750 nm, < 100 fs, 200 mW) illuminates the sample through a beam splitter (90:10, R:T) and a microscope objective, which also collects the reflected light. A set of long pass filters passes only the luminescence and Raman emitted light, which is directed to a spectrometer and an electron multiplying charge-coupled device (EMCCD) using a removable mirror. Without the mirror, light is directed to a Hanbury-Brown Twiss setup, which consists of a 50:50 beam splitter (BS) and two single photon avalanche diodes (SPAD) with lense (L) of f=60 mm. Inset shows a spectrum measured on the EMCCD.

Supplementary References

1. Chikkaraddy, R. *et al.* Mapping Nanoscale Hotspots with Single-Molecule Emitters Assembled into Plasmonic Nanocavities Using DNA Origami. *Nano Letters* **18,** 405–411 (2018).
2. Douglas, S. M. *et al.* Rapid prototyping of 3D DNA-origami shapes with caDNAno. *Nucleic Acids Res* **37,** 5001–5006 (2009).
3. Benz, F. *et al.* Generalized circuit model for coupled plasmonic systems. *Optics Express* **23,** 33255 (2015).
4. Plekhanov, A. I. & Shelkovnikov, V. V. Optical constants of nanofilms of J aggregates of organic dyes, measured by spectral ellipsometry and polarization reflectometry. *Opt. Spectrosc.* **104,** 545 (2008).
5. Mertens, J. *et al.* Tracking Optical Welding through Groove Modes in Plasmonic Nanocavities. *Nano Letters* **16,** 5605–5611 (2016).
6. Tserkezis, C. *et al.* Hybridization of plasmonic antenna and cavity modes: Extreme optics of nanoparticle-on-mirror nanogaps. *Physical Review A* **92,** (2015).
7. Loudon, R. *The quantum theory of light*. (Clarendon Press, 1973).
8. Fox, M. *Quantum Optics: An Introduction*. (Oxford University Press, 2006).
